# Identification of the role of SNARE proteins in rAAV vector production through interaction with the viral MAAP

**DOI:** 10.1016/j.omtm.2024.101392

**Published:** 2024-12-05

**Authors:** Cagla Aksu Kuz, Kang Ning, Siyuan Hao, Shane McFarlin, Xiujuan Zhang, Fang Cheng, Jianming Qiu

**Affiliations:** 1 Department of Microbiology, Molecular Genetics and Immunology, University of Kansas Medical Center, Kansas City, KS 66160, USA

**Keywords:** rAAV, SNARE, MAAP, vector secretion, vector production

## Abstract

Adeno-associated virus (AAV) expresses a membrane-associated accessory protein (MAAP), a small nonstructural protein, that facilitates AAV secretion out of the plasma membrane through an association with extracellular vesicles during AAV egress. Here, we investigated the host proteins that interact with AAV2 MAAP (MAAP2) using APEX2-mediated proximity labeling. We identified two SNARE proteins, Syntaxin 7 (STX7) and synaptosome-associated protein 23 (SNAP23), a vesicle (v-)SNARE and a target (t-)SNARE, respectively, that mediate intracellular trafficking of membrane vesicles aand exhibited associations with MAAP2 in HEK293 cells. We found that MAAP2 indirectly interacted with STX7 or SNAP23, and that the knockout of *STX7* or *SNAP23* not only enhanced rAAV secretion into the media but also increased total vector yield during rAAV vector production in HEK293 cells. Thus, our study revealed a practical approach for producing higher yields of rAAV vectors from the media, easing downstream processes in rAAV manufacturing.

## Graphical abstract

**Figure:**
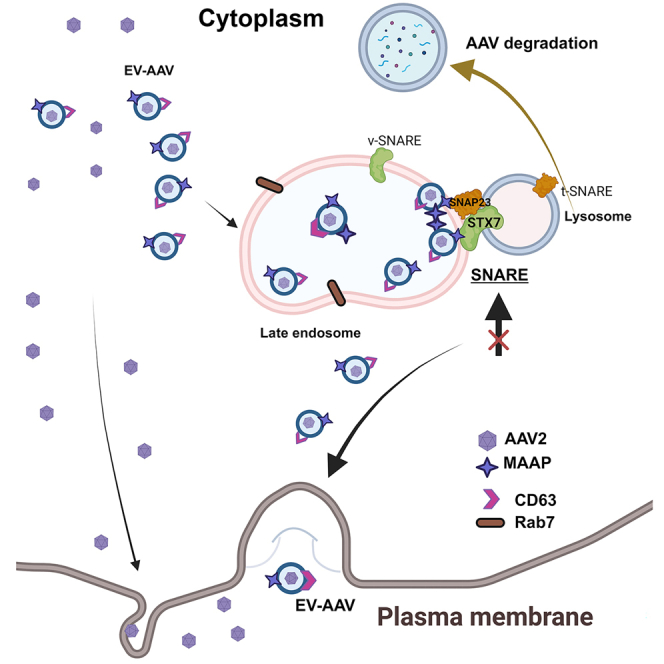

## Teaser

Qiu and colleagues identified that the membrane-associated accessory protein (MAAP) of AAV is associated with the SNARE complex. They also revealed that the knockout of the SNARE proteins, STX7 or SNAP23, enhances the secretion of AAV from the cells and increases the overall vector yield during vector production in HEK293 cells.

## Introduction

Adeno-associated viruses (AAVs) belong to the genus *Dependoparvovirus* within the family Parvoviridae. AAVs are single-stranded DNA (ssDNA) viruses that package an ∼4.7-kb viral genome within a non-enveloped T = 1 icosahedral capsid.^1^ The viral genome contains two major open reading frames (ORFs), *Rep* and *Cap*, positioned between two identical inverted terminal repeats (ITRs). The *Rep* ORF expresses two large (Rep78/68) and two small (Rep52/40) replication-essential nonstructural proteins.^2^^,^^3^^,^^4^^,^^5^ The *Cap* ORF encodes three structural proteins: VP1, VP2, and VP3 at a stoichiometric ratio of ∼1:1:10,^6^^,^^7^^,^^8^ and two small nonstructural/accessory proteins, i.e., assembly activating protein (AAP) and membrane-associated accessory protein (MAAP).^9^^,^^10^^,^^11^ Recombinant AAV (rAAV) has been widely utilized as a delivery vector in human gene therapy. To date, six rAAV-based human gene therapy medicines, Luxturna, Zolgensma, Elevidys, Roctavian, Hemgenix, and Beqvez,^12^^,^^13^^,^^14^^,^^15^^,^^16^^,^^17^ have been approved by the US Food and Drug Administration.

MAAP plays various roles in both wild-type (wt)AAV life cycle and rAAV production by facilitating AAV release from the plasma membrane.^11^^,^^18^^,^^19^ It negatively regulates viral DNA replication,^11^^,^^20^ and therefore, knockout or optimization of MAAP increases rAAV vector production.^11^^,^^20^ MAAP facilitates AAV egress through an association with extracellular vesicles (EVs).^18^ AAV2 MAAP (MAAP2) is predominantly localized in the cytoplasm, alongside the capsid, near the nuclear and plasma membranes, but the AAV5 MAAP (MAAP5) predominantly localizes within the nucleus.^11^ The exact mechanisms by which MAAP facilitates AAV egress through EVs remain unclear.^18^^,^^21^

In this study, we used an enhanced ascorbate peroxidase 2 (APEX2)-mediated proximity labeling to identify host proteins that interact with MAAP2. We identified that MAAP2 interacted with two SNARE (Soluble N-ethylmaleimide-sensitive factor attachment protein receptors) proteins: Syntaxin 7 (STX7) and Synaptosome associated protein 23 (SNAP23). SNARE proteins are generally classified as target (t-)SNAREs and vesicle (v-)SNAREs, which are found in a broad range of biological membranes, such as the plasma membrane, Golgi apparatus, endoplasmic reticulum, endosomes, lysosomes, and other cellular vesicles.^22^^,^^23^^,^^24^^,^^25^ They ensure the efficient transfer of cargoes by facilitating and regulating fusion events of vesicular membranes with target membranes. Importantly, SNARE proteins mediate trafficking between endosomes and phagosomes with other endosomes, lysosomes, the Golgi apparatus, the plasma membrane, and the endoplasmic reticulum.^26^ SNAP23, a t-SNARE, is required for lysosomal release,^27^ catalyzing endosomal fusion events,^28^^,^^29^ and mediating exocytosis and phagocytosis.^30^ STX7, a v-SNARE, is found mainly in late endosomes and lysosomes, but also in recycling endosomes, early endosomes, the plasma membrane, and phagosomes.^26^ It is required for the fusion of late endosomes with lysosomes and plays a pivotal role in the final steps of lysosomal biogenesis.^31^

Notably, in this study we showed that knockout of the *STX7* or *SNAP23* gene exhibited enhanced vector release into the media with an overall increase in the total vector yield during rAAV production in HEK293 cells. Thus, in addition to revealing MAAP2-associated host proteins, we underscored a promising strategy for elevating vector secretion into the cell culture media, simplifying rAAV vector purification procedure from culture media only.

## Results

### Identification of host proteins associated with MAAP2

**Figure 1:**
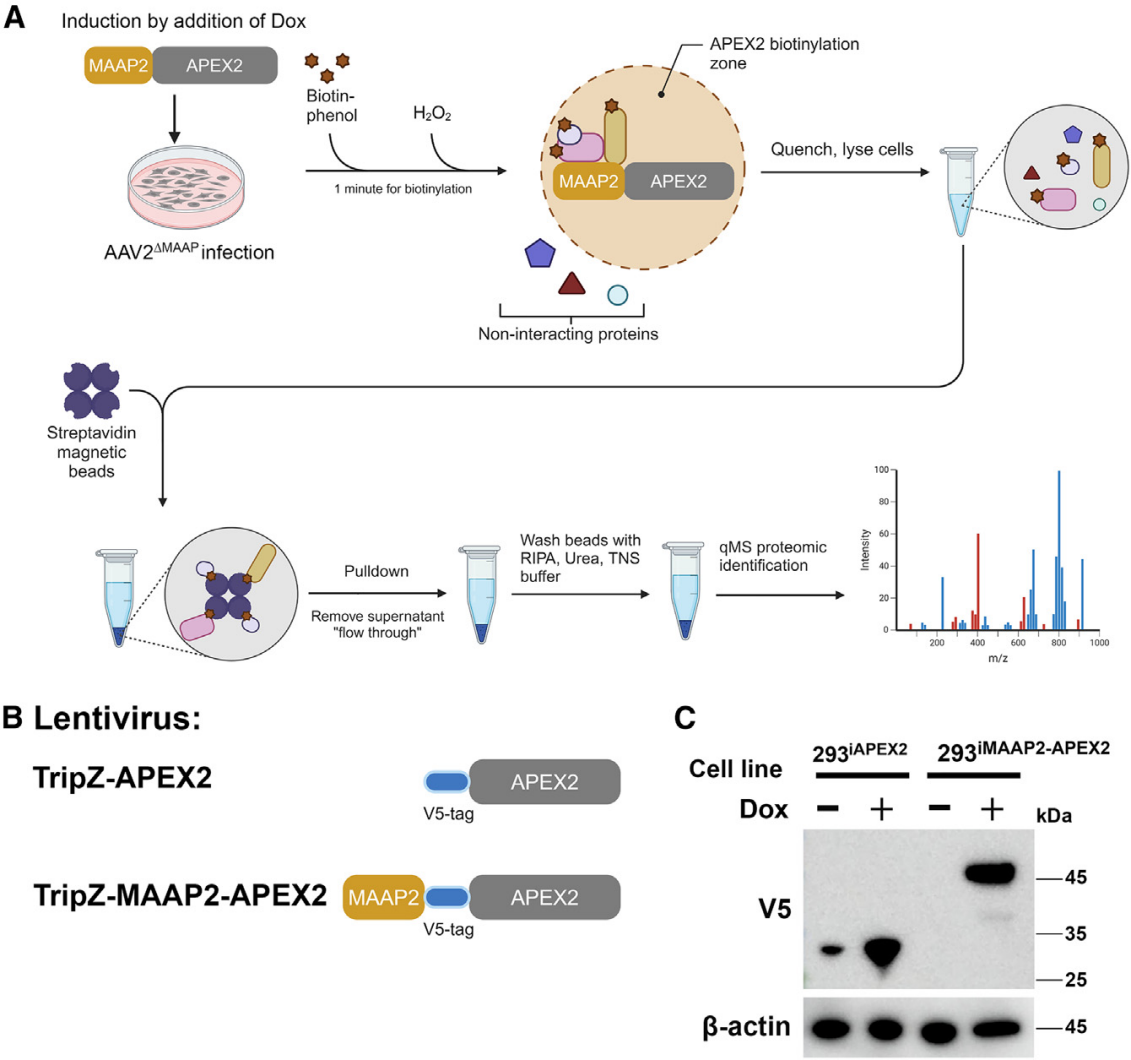
A workflow of APEX2-mediated proximity labeling and generation of inducible HEK293 cell lines expressing MAAP2-APEX2 and APEX2 (A) Diagram of workflow for APEX2-mediated proximity labeling. 293^iMAAP2−APEX2^ or 293^iAPEX2^ cells were infected with AAV2^ΔMAAP^ and transfected with pHelper. At 16 hpi, doxycycline (Dox) was added to induce expression of MAAP2-APEX2 or APEX2, and infected with AAV2^ΔMAAP^. Upon addition of biotin-phenol (BP) and H_2_O_2_, APEX2 catalyzes the oxidation of BP to biotin-phenoxyl radicals within ∼20 nm in diameter, thereby resulting in the biotinylation of MAAP2-associated proteins (APs) in 1 min. The reaction is then quenched and followed by a pulldown of MAAP2-APs using streptavidin magnetic beads. MAAP2-APs are identified by quantitative mass spectrometry (qMS). (B) MAAP2-APEX2 and APEX2 only expressing lentiviruses. TripZ-APEX2 and TripZ-MAAP2-APEX2 lentiviruses express APEX2 only (serves as negative control) and MAAP2-APEX2, respectively. APEX2 and MAAP2-APEX2 were expressed with a V5-tag at the N terminus of APEX2 as indicated. (C) Dox-inducible cell lines. 293^iAPEX2^ and 293^iMAAP2−APEX2^ cells were seeded on wells of a six-well plate, Dox was added to induce expression of APEX2 (∼29 kDa with the linker and V5 tag) or MAAP2-APEX2 (∼43 kDa with the linker and V5 tag). Cell lysates of mock (−) and Dox-added (+) 293^iAPEX2^ or 293^iMAAP2−APEX2^ cells were immunoblotted for V5 tag. β-actin is shown as a loading control.

As MAAP plays a role in AAV egress without a direct interaction with the capsid,^11^^,^^18^^,^^19^^,^^20^ we aimed to identify host proteins that interact with MAAP2 for a role in modulation of MAAP-related AAV capsid egress. To this end, we employed APEX2-mediated proximity labeling to identify the MAAP2-associating proteins (MAAP2-APs) in living cells.^32^ APEX2 is an engineered ascorbate peroxidase that is widely used for proximity biotinylation. APEX2 uses biotin-phenol (BP) as a substrate to biotinylate endogenous proteins found in close proximity (∼20 nm) in the presence of hydrogen peroxide (H_2_O_2_) by oxidizing BP into biotin-phenoxyl radicals (Figure 1A).^32^ The biotinylated proteome presents a snapshot of the cellular environment at a specific time point as this peroxidase reaction labels proximity proteins within ∼1 min.^33^^,^^34^^,^^35^

**Figure 2:**
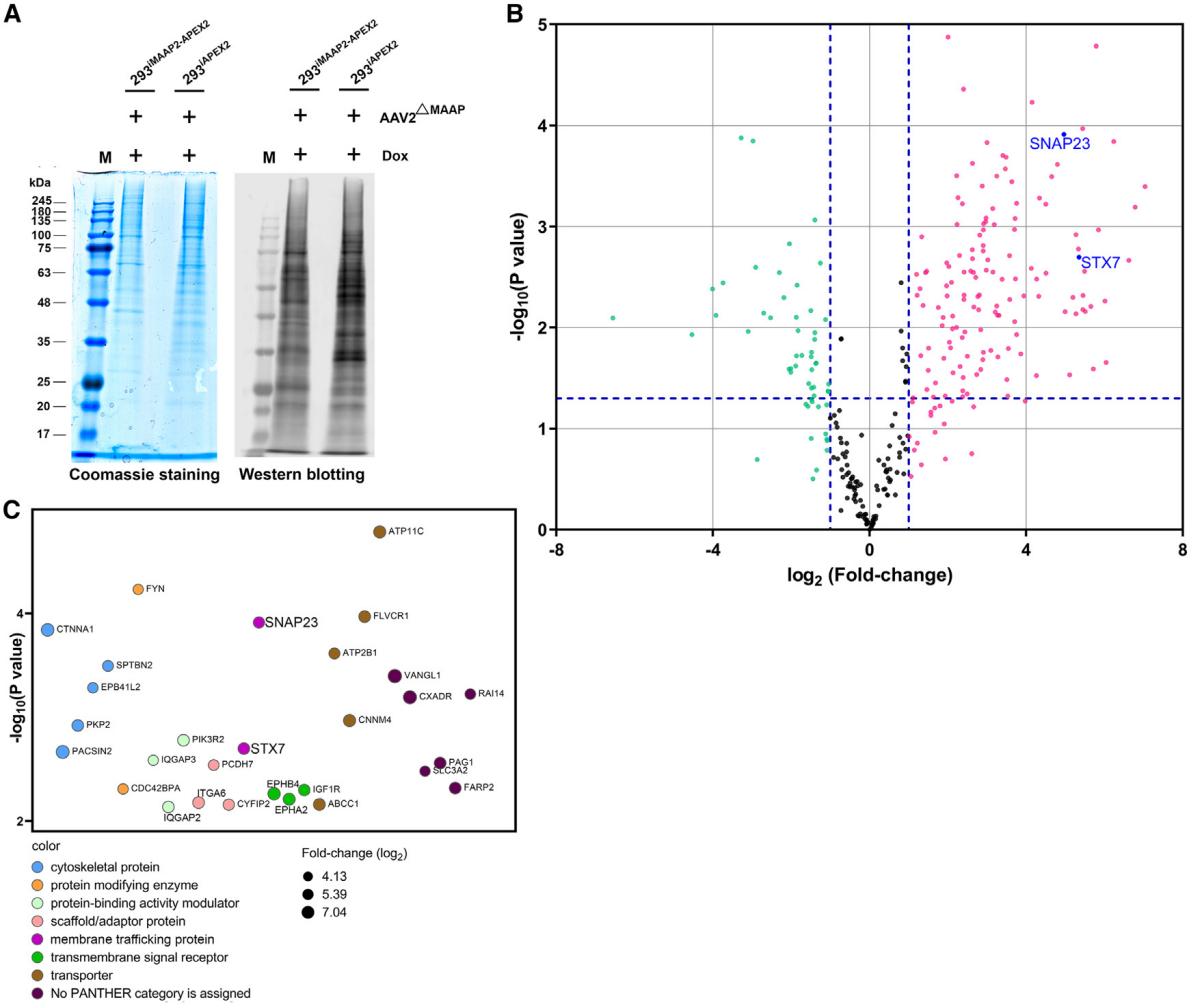
Identification of MAAP2-associating host proteins by APEX2-mediated proximity labeling (A) Optimized biotinylation for sampling of quantitative mass spectrometry (qMS). 293^iMAAP2−APEX2^ and 293^iAPEX2^ cells in a T25 flask were infected with AAV2^ΔMAAP^ at an MOI of 5,000 vgc/cell. At 16 hpi, Dox was added. At 2 dpi, APEX2-mediated biotinylation was carried out as described in materials and methods. Cells were incubated with biotin-phenol for 30 min at 37°C. H_2_O_2_ was added and incubated for 1 min at room temperature to biotinylate proximal proteins. The reaction was then quenched. The cells were harvested and lysed in RIPA buffer. The supernatant of the lysates was then incubated with streptavidin beads. Ten percent of the washed beads were separated on SDS-PAGE for Coomassie staining and western blotting (WB), respectively. The western blot was probed with Alexa Fluor 680-conjugated streptavidin (Thermo Fisher). (B) Analysis of the identified proteins by qMS. The remaining 90% of the washed beads were subjected to on-bead digestion and liquid chromatography-tandem mass spectrometry analysis. Three repeats were carried out. The qMS data listed in Table S1 were analyzed. The volcano plot shows statistical significance (−log_10_, *p* value) vs. the magnitude of change (log_2_, fold-change) for differentially interacted proteins between MAAP2-APEX2-and APEX2-expressing cells. Pink dots indicate enriched proteins by >2-fold and green dots indicate depleted proteins by >2-fold. The t test (unpaired, two-tailed) was employed for determination of statistical significance (*p* value). The horizontal dash line indicates *p* < 0.05. (C) Gene Ontology (GO) annotation of top enriched proteins. In the volcano plot (B), proteins clustered in the upper/right corner as divided by the blue dash lines, which were enriched by >16 times and had significance (*p* value) of >0.01 in the MAAP2-APEX2-expressing cells, were analyzed by GO. Proteins are shown in circles with colors indicating their functions. The size of the circles represents the enrichment score (fold-change in log_2_).

We first established two inducible HEK293 cell lines, 293^iMAAP2−APEX2^ and 293^iAPEX2^ (Figure 1B). Expression of MAAP2-APEX2 or APEX2 in the cell lines was induced to express at a similar level by the addition of doxycycline (Dox) (2 μg/mL) (Figure 1C). For APEX2-mediated proximity labeling, 293^iMAAP2−APEX2^ and 293^iAPEX2^ cells were infected with an MAAP mutant virus (AAV2^ΔMAAP^) with transfection of pHelper that expresses adenovirus helper genes, *E2a*, *E4orf6*, and *VA*.^11^ AAV2^ΔMAAP^-infected cells had Dox added at 16 h post-infection (hpi). At 48 hpi, MAAP2-APs in a diameter of ∼20 nm surrounding the MAAP2 in the infected cells were biotinylated upon the addition of BP and H_2_O_2_.^33^^,^^34^^,^^35^ The biotinylated MAAP2-APs were pulled down using streptavidin magnetic beads. Approximately 10% of the pulldown proteins were analyzed by SDS-PAGE followed by Coomassie blue staining and western blotting probed by streptavidin (Figure 2A). Approximately 90% of the pulldown proteins (beads) were subjected to on-bead digestion and quantitative mass spectrometry (qMS). The qMS results identified 319 proteins with a sum of unique reads of 15 or more upon MAAP2-APEX2 expression (Figure 2B; Table S1). The highly enriched proteins of −log_10_ *p* > 2 and log_2_ >4 were categorized using Gene Ontology (GO) analysis to address the proteins involved in intracellular trafficking. Two membrane trafficking proteins, STX7 and SNAP23, were identified (Figure 2C).

Taken together, analysis of data obtained from APEX2-mediated proximity labeling coupled with qMS revealed MAAP2-APs in MAAP-APEX2-inducible HEK293 cells infected with AAV2^ΔMAAP^. STX7 and SNAP23, GO-categorized by membrane trafficking function, were enriched upon MAAP2-APEX2 expression, and importantly STX7 and SNAP23 appear presenting in the SNARE complex as a v-SNARE and a t-SNARE, respectively.^24^^,^^25^^,^^36^ Therefore, we chose STX7 and SNAP23 for further investigation.

### STX7 or SNAP23 indirectly interacts with MAAP2

**Figure 3:**
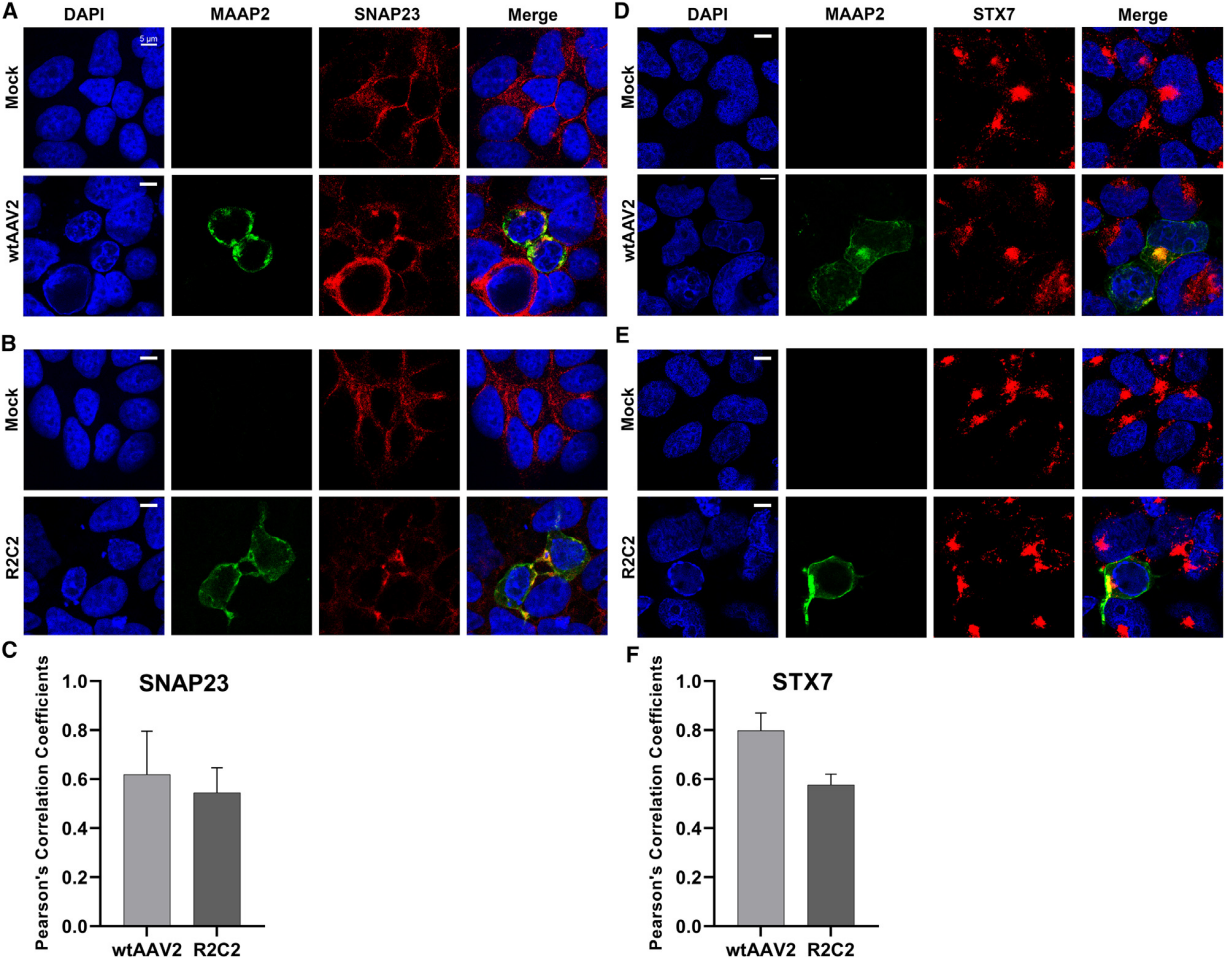
STX7 or SNAP23 colocalizes with MAAP2 in both wtAAV2-infected cells and rAAV2-producing cells (A and D) wtAAV2 infection. HEK293 cells were mock-infected or infected with wtAAV2 followed by pHelper transfection. At 2 dpi, the cells were co-immunostained for MAAP2 and SNAP23 (A) or MAAP2 and STX7 (C). (B and E) rAAV2 production. HEK293 cells were mock or transfected with pR2C2, pHelper, and prAAV2. At 2 dpt, the transfected cells were co-immunostained for MAAP2 and SNAP23 (B) or MAAP2 and STX7 (D). SNAP23 (B) or STX7 (D) cells were co-immunostained with a secondary antibody conjugated with a far-red dye. Co-immunostained cells were observed under a Leica STED microscope with a 100× objective lens. Images captured in the far-red wavelength were pseudo-colored in red. The colors of confocal images correspond to blue for DAPI, green for MAAP, and red for SNAP23 or STX7 as indicated. Scale bar, 5 μm. Representative confocal images are shown. (C and F) Quantification of colocalization. Pearson’s correlation coefficients were measured for colocalization of MAAP2 with SNAP23 (C) or STX7 (F) using NIH ImageJ.

We performed immunofluorescence analysis to confirm the association of MAAP2 with STX7 or SNAP23 in wtAAV2-infected and rAAV2-producing HEK293 cells, respectively. HEK293 cells were infected with wtAAV2 followed by pHelper transfection. For rAAV2 production, HEK293 cells were transfected with pR2C2, pHelper, and prAAV2. At 2 days post-infection (dpi), the infected cells were first stained with a cell membrane tracking dyes (MemBrite, Biotium) as well as with anti-MAAP2 and anti-AAV2 capsid antibodies. We found MAAP2 was costained with the cell membrane tracking dye, as well as with some capsids (Figure S1), confirming that MAAP2 is plasma membrane-associated as previously suggested.^10^^,^^11^^,^^18^^,^^19^ Moreover, at 2 dpi or 2 days post-transfection (dpt), the cells were co-immunostained for MAAP2 and STX7 or SNAP23. The results showed that SNAP23 colocalized with MAAP2 in the cytoplasm near the plasma membrane in both infected and transfected cells (Figures 3A–3C). Similarly, the colocalization of STX7 with MAAP2 was observed in the cytoplasm mostly near the plasma membrane of both infected and transfected cells (Figures 3D–3F).

**Figure 4:**
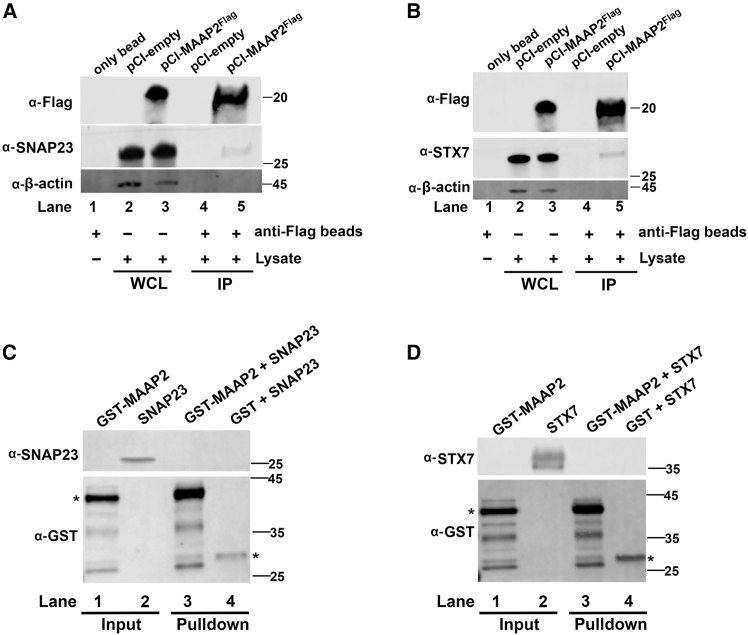
SNAP23 or STX7 interacts with MAAP2 in cells but not *in vitro* (A and B) Co-IP. HEK293 cells were transfected with pCI-MAAP2^Flag^ or pCI-empty. At 2 dpt, cells were harvested and lysed. 90% of the lysates were used for immunoprecipitation with anti-Flag-conjugated magnetic beads. Western blotting was performed for detection of MAAP2^Flag^ and SNAP23, respectively (A), and for detection of MAAP2^Flag^ and STX7, respectively (B). β-actin is shown as a loading control. 10% of the lysates were loaded as the whole cell lysate (WCL). (C and D) *In vitro* pulldown assay. Approximately 2 μg of the purified GST-MAAP2 protein and negative control GST protein were employed as baits to pull down ∼2 μg prey proteins; purified SNAP23 (C) or STX7 (D) using glutathione agaroses. Western blotting was performed for detection of GST-MAAP2 and control GST using anti-GST (C and D), for the detection of SNAP23 using anti-SNAP23 (C), and for the detection of STX7 using anti-STX7 (D). ∼200 ng of the bait and pray proteins were loaded as inputs. Asterisks indicate the major detected GST-MAAP and GST.

Next, we performed co-immunoprecipitation (co-IP) to investigate the interactions between MAAP2 and STX7, as well as between MAAP2 and SNAP23. To this end, HEK293 cells were mock-transfected with pCI-empty plasmid or transfected with pCI-MAAP2^Flag^. At 2 dpt, cell lysates were processed to pull down the MAAP2-interacting proteins using anti-Flag magnetic beads, and immunoblotted with the respective antibodies (Figures 4A and 4B). The results showed a specific band of SNAP23 or STX7 was detected in both the samples pulled down by MAAP2^Flag^ (Figures 4A and 4B, lane 5). Thus, the co-IP results confirmed an interaction of MAAP2 with SNAP23 or STX7. To further examine whether these interactions are direct, we performed *in vitro* pulldown assay using purified SNAP23 or STX7 protein. We purified a GST-tagged MAAP2 (GST-MAAP2) protein and employed it as a bait to pull down the purified prey protein, STX7 or SNAP23. The results showed that neither STX7 nor SNAP23 was pulled down by GST-MAAP2 (Figures 4C and 4D, lane 3).

Collectively, we verified that two membrane trafficking proteins, STX7 and SNAP23, indirectly interact with MAAP2 during both wtAAV2 infection and rAAV2 production. However, no direct interaction between MAAP2 and STX7 or SNAP23 was observed *in vitro*. We speculate that MAAP2 is spatially closely associated with STX7 or SNAP23 in the same cellular compartments in both wtAAV2-infected and rAAV2-producing cells through intermediator/protein(s) or close localization on the same intracellular membrane vesicles.

### Knockout of *STX7* or *SNAP23* expression results in higher vector release into the media of rAAV-producing cells

**Figure 5:**
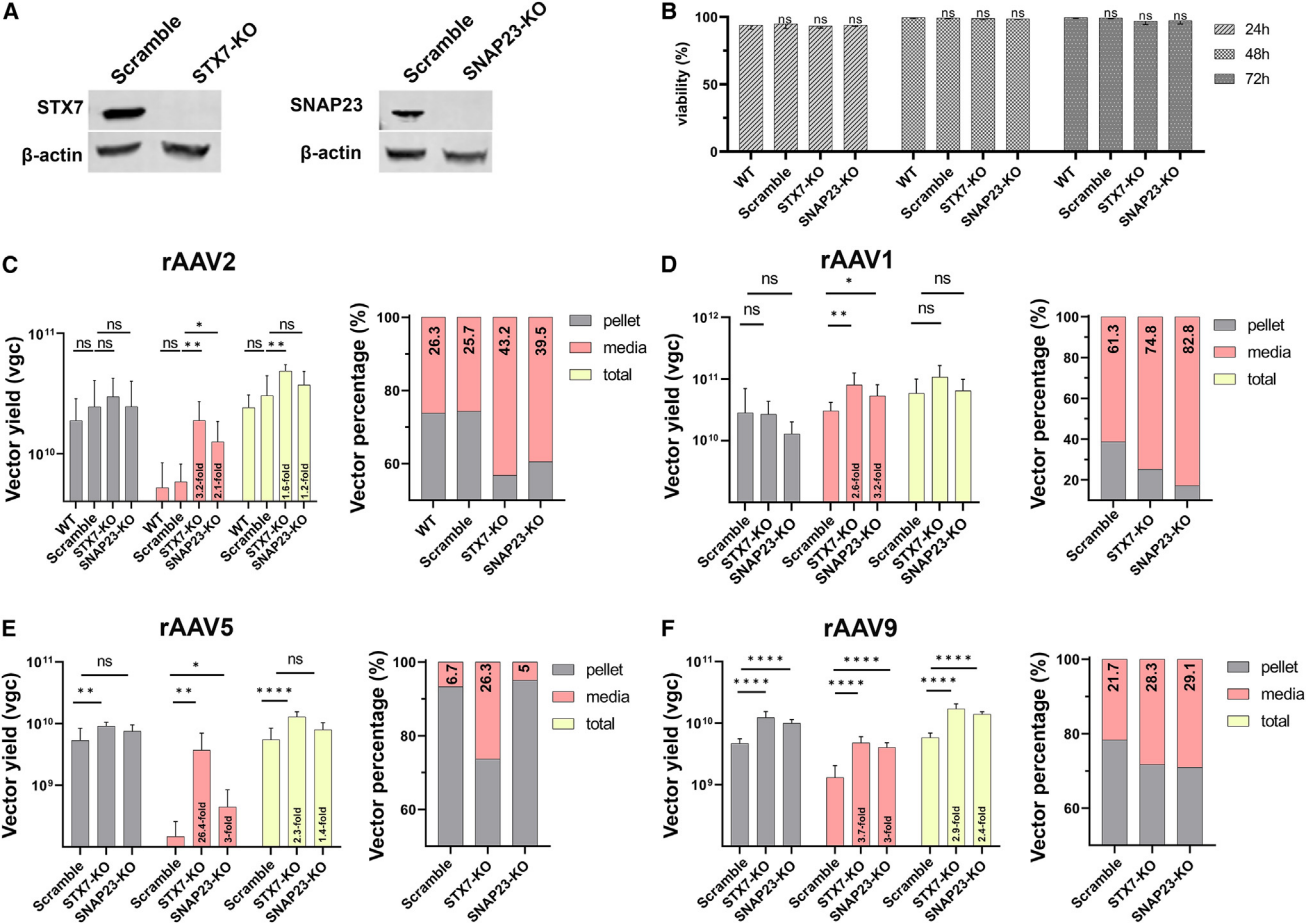
Knockout of *STX7* or *SNAP23* increases rAAV vector secretion into the media, as well as total vector yields, during rAAV production (A and B) Generation of KO cell lines. (A) Western blotting. HEK293 cells were transduced with lentiviruses expressing *STX7*-, *SNAP23*-targeting, or scramble guide RNA (Scramble), followed by single-cell cloning for STX7-KO cell line generation. SNAP23-KO cell line was not single-cell cloned. Cells were analyzed for expression of STX7 or SNAP23, as indicated, using western blotting. β-actin serves as a loading control. (B) Cell viability. WT, Scramble, STX7-KO, and SNAP23-KO HEK293 cells were seeded in wells of six-well plates at equal cell number. Cells were trypsinized, and the viable cells were counted after staining with trypan blue at 24, 48, and 72 h, respectively. (C–F) Small-scale production of rAAV2 (C), rAAV1 (D), rAAV5 (E), and rAAV9 (F) in the gene knockout cells. WT, Scramble, STX7-KO, and SNAP23-KO HEK293 cells, as indicated, were transfected with pR2C2 (C), pR2C1 (D), pR2C5 (E), or R2C9 (F), together with pHelper and prAAV2. At 3 dpt, cells and media were harvested for subsequent treatments. DNase-digestion-resistant viral DNA was extracted from crude lysates of harvested cells (pellet) and media, respectively. Produced rAAV vectors in the pellet and media were quantified by qPCR using an mCherry probe. Left panel: Related bars represent total vector yields in vector genomic copies (vgc) in the pellet (cells), media and total (pellet plus media), respectively. Right panel: Bars indicate ratios of produced rAAV yield in the pellet (cells) vs. in media based on the data presented in the left panel. Means and SDs were calculated using data from three independent experiments (*n* = 3). ∗*p* < 0.05; ∗∗*p* < 0.01; ∗∗∗∗*p* < 0.0001; and ns, no significant difference.

As MAAP plays a role in the release of AAV from the cell plasma membrane (egress), we examined the role of STX7 and SNAP23 in rAAV production. To this end, we knocked out *STX7* or *SNAP23* in HEK293 cells by using CRISPR-Cas9 technique. The knockout of *STX7* or *SNAP23* in HEK293 cells and scramble guide RNA (gRNA)-expressing control cells (Scramble) were confirmed by immunoblot analysis (Figure 5A), and did not have any effect on cell viability, compared with parent (WT) HEK293 cells (Figure 5B).

Next, we produced several serotypes of rAAV representing various AAV capsid clades in WT, Scramble, STX7-KO, and SNAP23-KO HEK293 cells, respectively. The cells were transfected with a corresponding Cap-expressing pRepCap plasmid, along with pHelper and prAAV2 for rAAV production on a small scale. WT and Scramble HEK293 cells did not show a significant difference in rAAV2 production (Figure 5C). Thus, other serotypes were produced only in Scramble, STX7-KO, and SNAP23-KO HEK293 cells. Notably, in all tested serotypes, STX7-KO and SNAP23-KO cells led to not only more vector released in the media but also a higher total vector yield than those from the Scramble (control) cells, but the observed increases were more significant in the STX7-KO cells (Figures 5C–5F).

In rAAV2 production, the total vector produced in STX7-KO and SNAP23-KO cells were 1.6- and 1.2-fold more, respectively, than those in the Scramble cells (Figure 5C, left panel). Strikingly, the increases correspond to a vector release of ≥40% in the media of STX7-KO and SNAP23-KO cells, compared with a 26% vector release from the Scramble cells (Figure 5C, right panel). As rAAV1 naturally has more vectors being released to the media than others,^37^ the Scramble control showed the highest percentage (61.3%) of the secreted vectors among all tested serotypes. Nevertheless, rAAV1 vectors released from the STX7-KO and SNAP23-KO 293 cells were further increased to ∼75% and ∼83%, respectively (Figure 5D, right). However, the total vector yields of rAAV1 in both KO cells were not significantly increased (Figure 5D, left).

Remarkably, the number of secreted vectors of rAAV5 in the media was ∼4 times higher from the STX7-KO cells compared with the Scramble cells, equating to ∼26% of the vector release in the media, compared with the only 6.7% from the Scramble control cells (Figure 5E, right). However, there was no increase in vector release of the SNAP23-KO cells. There were also slight increases in the total vector yields from both KO cells (2.3- and 1.4-fold for SXT7-KO and SNAP-KO, respectively) (Figure 5E, left). A similar tendency was observed in rAAV9 production. Not only vector release but also total yield exhibited an increase in both STX7-KO and SNAP23-KO cells. The number of the vectors in the media of STX7-KO and SNAP23-KO cells were increased by ∼7% compared with the 21.7% from the Scramble cells (Figure 5F, right). The total yields of rAAV9 produced in STX7-KO cells and SNAP23-KO cells were 2.9 and 2.4 times higher, respectively, compared with those from the Scramble cells (Figure 5F, left).

During AAV2 infection, we also observed that SNAP23-KO cells had an increase in the release of progeny virions (∼75% in the media of SNAP23-KO vs. 60% in WT HEK293 cells) (Figure S2B). However, infection of the MAAP KO mutant virus, AAV2^ΔMAAP^, in SNAP23-KO cells released only ∼1% virions in the media, and *SNAP23* KO resulted in a significant but slight increase (to ∼2%) in virion secretion (Figure S2C). These observations supported that MAAP is required for the egress of progeny virions into the media.^11^

Collectively, both *S*TX7-KO and SNAP23-KO cells exhibited an enhanced secretion of rAAV2, rAAV1, rAAV5, or rAAV9 vector into the media by 7%–20% (except for the rAAV5 in SNAP23-KO cells), which resulted in an increase of >2-fold in vector secretion into the media (Figures 5C–5F, left). In addition, STX7-KO cells produced a significantly high total yield (>1.6-fold) of rAAV2, rAAV5, and rAAV9. These results suggest that STX7 and SNAP23 play a negative role in the release/secretion of rAAV vectors from the triple-plasmid transfected HEK293 cells, and that rAAV release is dependent on the function of MAAP.

### Large-scale production of rAAV5 in STX7-KO and SNAP23-KO HEK293 cells

**Figure 6:**
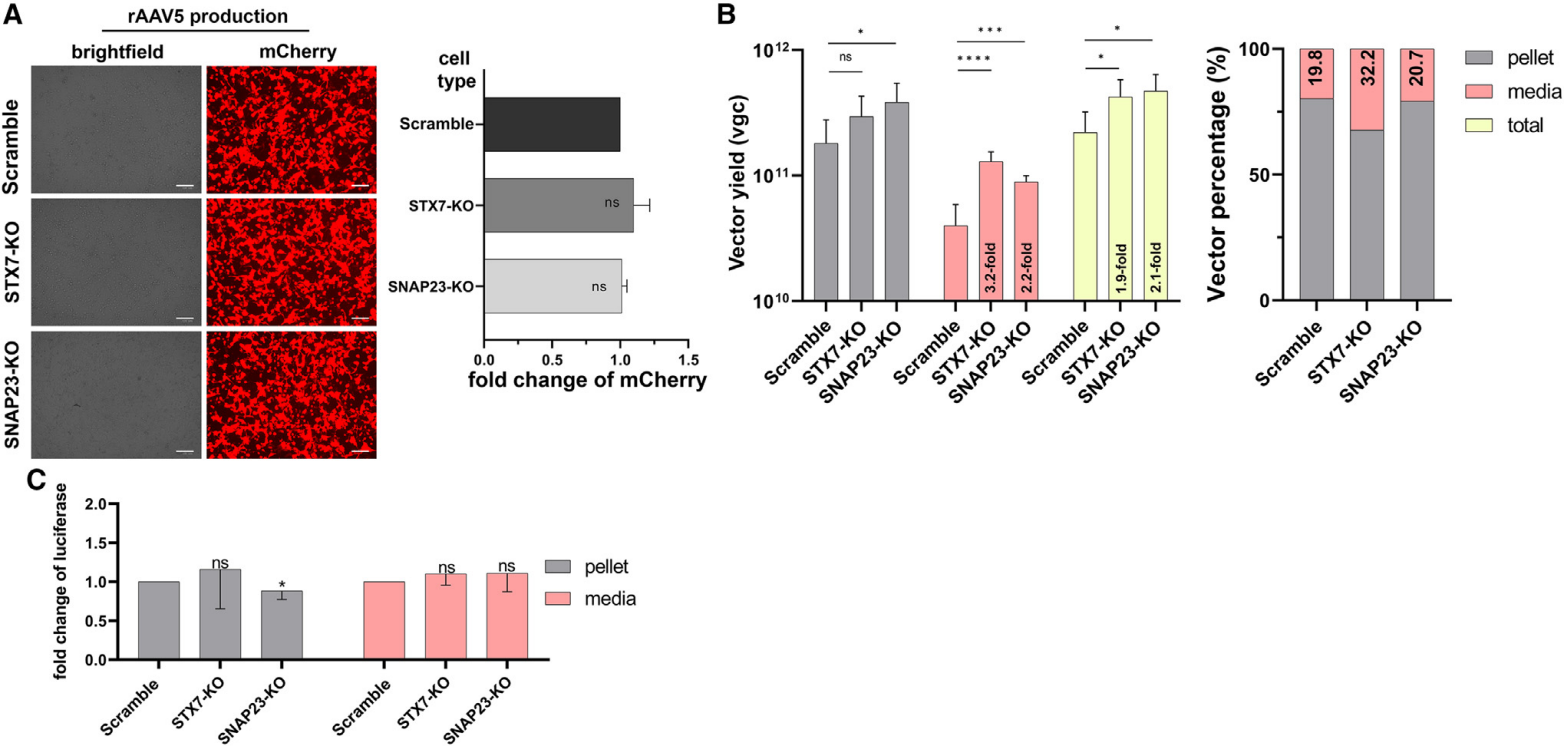
Large-scale production of rAAV5 in STX7-KO and SNAP23-KO HEK293 cells Scramble, STX7-KO, and SNAP23-KO HEK293 cells were transfected with pR2C5, pHelper, and prAAV2. (A) mCherry expression. Transfected cells were imaged for mCherry expression at 2 dpt by the ZOE Fluorescent Cell Imager (Bio-Rad). Representative images are shown. The intensity of mCherry expression of each group was displayed as fold changes relative to scramble control group. The relative fold changes of mCherry expression in the transfected cells were measured by ImageJ. (B) Titers of the purified rAAV5 vectors in the cells (pellet) and in the media. At 3 dpt, cells and media were harvested for vector purification. DNase-digestion-resistant viral DNA were extracted from the final purified vectors from the pellets and media and quantified by qPCR. Left panel: Data shown are total vector yields (vgc) in pellet, media or total (pellet plus media). Right panel: Bars indicate ratios of produced rAAV yield in the pellet (cells) vs. in the media based on the data presented in the left panel. (C) Transduction efficiency. HEK293 cells were transduced with rAAV5 purified from the cells and the media of the indicated HEK293 cell lines (x axis). Bars represent the intensity of luciferase activity of each group, shown as fold changes relative to the Scramble group (y axis). Means and SDs were calculated using data from three independent experiments (*n* = 3). ∗*p* < 0.05; ∗∗∗*p* < 0.001; and ∗∗∗∗*p* < 0.0001; and ns, no significant difference.

We further produced rAAV5 at a large scale in the gene knockout cells, as the small-scale production of rAAV5 displayed the highest vector secretion into the media in STX7-KO cells. To this end, Scramble, STX7-KO, and SNAP23-KO cells were transfected with pR2C5, pHelper, and prAAV2 in 150-mm dishes. The transfection efficiency in each cell line was evaluated by measuring mCherry expression at 2 dpt. STX7-KO and SNAP23-KO cells displayed no significant difference in the mCherry expression, compared with the Scramble cells (Figure 6A). rAAV5 vectors purified by CsCl ultracentrifugation from either pellets or media of the transfected Scramble, STX7-KO, and SNAP23-KO cells exhibited >95% full-particles as observed under a transmission electron microscope. The large-scale vector production confirmed that STX7-KO cells had a significantly higher percentage (∼32%) of the vectors secreted in the media, compared with the ∼20% from the Scramble cells (Figure 6B, right). The total yields of purified rAAV5 were significantly higher, 1.9 and 2.1 times higher in STX7-KO and SNAP23-KO cells, respectively, than that in the Scramble control cells (Figure 6B, left). Again, as observed in the small-scale production, the relative level (%) of the vector released to the media from SNAP23-KO cells was not increased. The purified vectors from the media or cell pellets of either STX7-KO or SNAP23-KO cells exhibited no significant difference in transduction efficiency (Figure 6C).

Taken together, the effect of *STX7* KO in rAAV5 production was applicable in large-scale production with no difference in the transduction efficiency of the produced vectors. Our results demonstrated that the STX7-KO HEK293 cell line significantly enhances rAAV5 secretion via the plasma membrane and total vector production, making it valuable for large-scale rAAV5 production applications.

## Discussion

In this study, we identified two important SNARE proteins, STX7 and SNAP23, that indirectly interact with MAAP2. They colocalize near the plasma membrane in rAAV-producing cells, as well as in wtAAV-infected cells. *STX7* or *SNAP23* KO significantly increases rAAV vector secretion via the plasma membrane. The enhancement applies to various AAV serotypes, including AAV1, 2, 5, and 9 in various clades of AAVs. Except for rAAV5 in *SNAP23* KO cells, *STX7* or *SNAP23* KO increases rAAV vector secretion via the plasma membrane by 7%–20%. *STX7* KO also significantly increases the total vector production by 2- to 3-fold. Thus, our findings provide a practical approach to increase AAV vector yield produced in the media of the triple-plasmid-transfected HEK293 cells, which could simplify the process of rAAV vector purification significantly.

**Figure 7:**
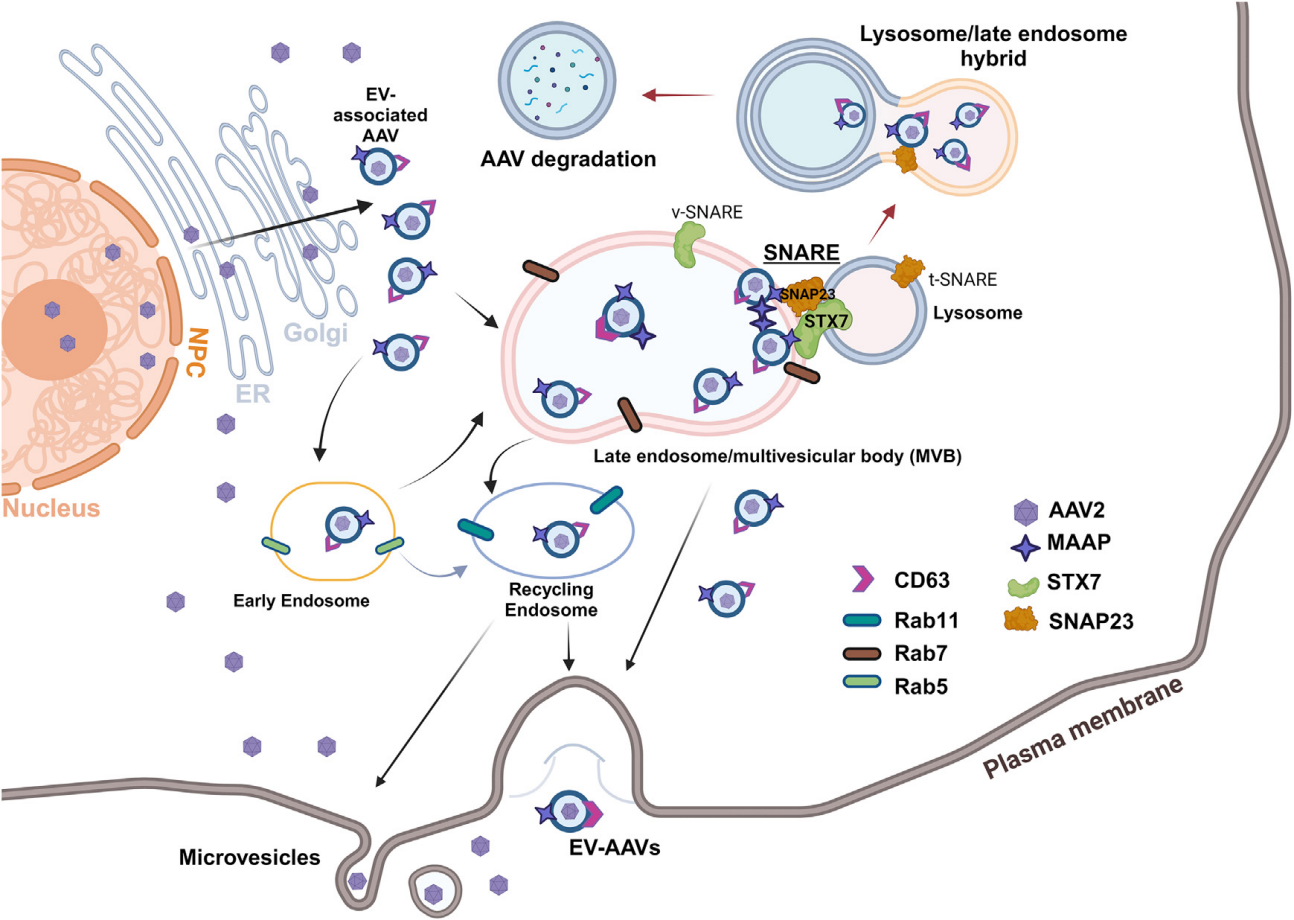
A proposed model of the role of SNARE proteins in AAV egress AAV capsids produced in the nucleus are egressed through the nuclear pore complex (NPC), the endoplasmic reticulum (ER), and the Golgi apparatus, where a portion of the vectors are associated with EVs (marked with exosome marker CD63), on which MAAP2 is attached.^18^^,^^21^ These EV-associated or -carried AAVs are routed to late endosomes (Rab7+) or MVB through the egress pathway of EVs as exosomes to secrete EV-AAVs. A portion of the late endosomes/MVBs are captured or recognized by v-SNARE proteins (e.g., STX7). STX7 interacts with t-SNARE proteins, e.g., SNAP23, for the fusion of late endosomes with lysosome,^46^ where SNAP23 makes up the heterodimeric t-SNAREs required for lysosome exocytosis, leading to the formation of lysosome/endosome hybrid for degradation of the virions. On the other hand, the EV-associated AAV can reach early endosomes (Rab5+) and then traffic to recycling endosomes (Rab11+), where they can be released as microvesicles.^21^ Knockout of SNARE expression can block the formation of a lysosome/endosome hybrid, resulting in less virion degradation and more virion release/secretion out of the plasma membrane.

Viruses hijack cellular machinery to facilitate virus entry, replication, and egress. Endosomal vesicles are targeted by viruses to initiate infection, transport viral components to the appropriate locations for genome replication, and afterward they are loaded with progeny viruses and traffic to the plasma membrane for release. rAAV has the nature to be released outside of the plasma membrane into the media either freely or associated with/inside extracellular vesicles (EVs), in particular exosomes^18^^,^^38^ (Figure 7). Secreted rAAV vectors were observed to be associated with EVs marked with CD81, CD63, and CD9,^18^ and secreted wtAAVs were also associated with exosome makers CD81 and CD63 (Figure S3). The EV-associated rAAV vectors only had a portion existing in the exosomes (<10%) of the media, and *STX7* or *SNAP23* KO did not increase the vector load in the exosomes (data not shown), arguing that other formats of EVs, in addition to the exosomes, may also contribute to the secretion of the vectors (Figure 7). Thus, the mechanisms underlying AAV egress remain elusive,^21^ and further investigation is necessary; however, the discovery of rAAV release into media through EVs^18^^,^^38^ garnered significant interest in the field and brought new insights into rAAV production and transduction. EV-packaged rAAV (EV-rAAV) has the ability to enhance transduction, provides efficient evasion from neutralizing antibodies, and broadens tissue tropism, including the central nervous system and lungs.^38^^,^^39^^,^^40^^,^^41^^,^^42^ Importantly, EV-rAAV can simply be purified from the culture medium of the triple-plasmid transfected HEK293 cells,^43^^,^^44^^,^^45^ highlighting the importance of rAAV secretion into the cell culture media.

Recent studies have shown that MAAP facilitates wtAAV egress in non-lytic infection and rAAV production through an association with EVs.^11^^,^^18^^,^^20^ MAAP plays a role in facilitating the association of AAV capsid with EVs for efficient release out of the plasma membrane.^18^ However, the specific pathways involved in these processes are obscure.^21^ Here, we showed that MAAP2 indirectly interacts with two SNARE proteins, SNAP23 and STX7. Although an indirect interaction of MAAP5 with SNAP23 or STX7 was not detected in co-IP, an association of the MAAP5 with SNAP23 or STX7 was observed in an immunofluorescence assay (data not shown). These indirect interactions or associations negatively regulate AAV vector release. This finding suggests that SNARE complexes are not involved in AAV egress; instead, they likely play a role in vector degradation, as *SNAP23* or *STX7* KO largely increases vector release into the media and the total vector yield.

SNARE proteins function in membrane vesicle trafficking and biological membrane fusion.^22^^,^^23^ SNAP23 is a t-SNARE, whereas STX7 is a v-SNARE.^24^^,^^25^^,^^36^ SNAP23 is found in vesicular membranes (as target vesicles) and the plasma membrane, and has a role in lysosomal secretion.^47^^,^^48^^,^^49^^,^^50^ STX7 is a v-SNARE, found in the late endosome, that plays a role in the fusion of those by forming a complex with its partners.^31^^,^^51^^,^^52^ STX7 also promotes autophagolysosome formation (fusion of autophagosome-lysosome)^53^ and depletion of STX7 blocks autophagic flux.^54^

In this study, we showed that SNAP23 colocalized with MAAP close to the plasma membrane, but the interaction was indirect. The depletion of SNAP23 resulted in increased viral release into the media in all tested AAV serotypes (except for AAV5) during rAAV production. We noted here that MAAP is colocalized and immunoprecipitated with STX7 or SNAP23 by an indirect interaction. Based on the increased release of rAAV in both SXT7-KO and SNAP23-KO cell lines, both STX7 and SNAP23 have a negative impact on vector secretion into the media. As SNAP23, STX7, and VAMP8 can form an SNARE complex and play a role in phagosome formation,^46^ we propose that the SNARE complex leads late endosomes to be targeted by the lysosome, resulting in endosome-lysosomal fusion as a late endosome/lysosome hybrid, in which rAAV vectors are degraded (Figure 7). It should be noted that wtAAV infection induces autophagy^55^^,^^56^; thus, it may be possible that during rAAV production, the autophagosome is formed and targeted by the lysosome through SNARE complex formation to mature autophagolysosome for AAV degradation.^57^ However, whether EV-associated AAV is targeted by the autophagolysosome is unknown. Apparently, further investigations are warranted to understand the function of the SNARE complex and whether autophagolysosome plays a role in rAAV vector biogenesis in the cytoplasm of the vector-producing cells.

Other than SNAP23 and STX7, SLC3A2 (Solute carrier family 3 member 2) and PACSIN2 (Protein kinase C and casein kinase substrate in neurons 2) are also some of the highly enriched proteins in qMS analysis. SLC3A2 (also known as CD98hc) is a transmembrane protein that functions as a chaperone for the membrane trafficking of amino acids and plays a role in cell growth, survival, and migration.^58^ PACSIN2 plays a role in the modulation of autophagy and endocytic trafficking.^59^ Its depletion resulted in HIV (human immunodeficiency virus) secretion,^60^ suggesting a possible role of PACSIN2 for viruses to hijack for spreading/release.^61^ Although we did not examine the function of PACSIN2 and SLC3A2 in the given scope, further investigation of these proteins could broaden our understanding of the mechanisms underlying how the MAAP is loaded onto/into EVs and the EV-associated AAV egress pathways.

In summary, we demonstrated depletion of STX7 or SNAP23 increased the release of rAAV1, rAAV2, rAAV5, and rAAV9 into media during rAAV production, which was accompanied by an overall enhanced rAAV vector yield. The vector increases of either in the media or overall is most likely due to the disrupted cellular function of the SNARE complex in routing the MAAP/EV-associated vectors through late endosome (Rab7^+^) to fuse with lysosomes for degradation of AAV vectors.^18^^,^^46^^,^^62^ We believe that knockout of the SNARE complex is a promising approach for optimizing downstream processes in rAAV manufacturing, as it results in a higher yield of vectors released in the media. Nevertheless, understanding the mechanisms underlying AAV egress out of the nucleus and plasma membrane as well as host proteins that interact with MAAP would shed light on novel approaches to simplify and enhance rAAV vector production.

## Materials and methods

### Cells and cell culture

Human embryonic kidney (HEK)293 cell line (293AAV) was purchased from Cell Biolabs, Inc. (San Diego, CA). HEK293 cells were maintained in Dulbecco’s modified Eagle’s medium (DMEM) (#SH30022; Cytiva Life Science, Marlborough, MA) supplemented with 10% fetal bovine serum (FBS; #F0926, MilliporeSigma, St. Louis, MO) and 100 units of penicillin-streptomycin. 293FT cells (ThermoFisher Scientific Inc, Waltham, MA) were maintained in DMEM with the addition of 10% FBS, 0.1 mM non-essential amino acids (MEM-NEAA; #25-025-CI, Corning Life Sciences, Corning, NY), and 6 mM L-glutamine (GlutaMAX; # 35050061, Thermo Fisher).

### Plasmid construction

#### pCI plasmids

pCI-empty and pCI-MAAP^Flag^ plasmids were constructed as described previously.^11^ pCI-MAAP2-APEX2 was constructed by cloning a codon-optimized MAAP2-APEX2 open reading frame (ORF) in pCI-Neo (Promega, Madison, WI) at GenScript (Piscataway, NJ). A seven-residue G-S (glycine-serine; GGSGGSG) linker and a V5-tag were inserted between MAAP2 and APEX2 ORFs.

#### pTripZ plasmids

pTripZmCherry,^63^ which is a lentiviral vector expression plasmid used for doxycycline-inducible gene expression, was used to clone MAAP2-APEX2 and APEX2 ORFs (from pCI-MAAP2-APEX2), respectively, by replacement of the mCherry ORF using HiFi assembly (NEB, Ipswich, MA), resulting pTripZ-MAAP2-APEX2 and pTripZ-APEX2.

#### pLentiCRISPRv2 plasmids

Three guide RNAs (gRNAs) targeting the *STX7* or *SNAP23* gene were individually cloned into plentiCRISPRv2 (#52961, Addgene, Watertown, MA). Sequences of *STX7*-targeting gRNAs were (1) 5′-GCG GGG TCA CCA CCA ACT CC, (2) 5′-CTC TCT CAT GAA TAA GAC GG-3′, and (3) 5′-GGA TGT TAG AAG AGA TCC TC-3′. Sequences of *SNAP23*-targeting gRNAs were (1) 5′- TGT TCA TCC AGC ATA GTG A-3′, (2) 5′-ATT ACA TGG GCA GAC ACA A-3′, and (3) 5′- CAG AAC TCA ACA AAT GCT G-3′. Scramble gRNA sequence (5′-GTA TTA CTG ATA TTG GTG GG-3′) was cloned into plentiCRISPRv2 as a scramble gRNA control.^64^^,^^65^

#### Plasmids for rAAV vector production

pAAV2RepAAV1Cap (pR2C1^66^), pAAV2RepCap (pR2C2), pAAV2RepAAV5Cap (pR2C5), pAAV2RepAAV9Cap (pR2C9/pXR9^67^) plasmids, adenoviral helper genes-containing plasmid, pHelper, and the rAAV2 genome plasmid, prAAV2 (pAV2F5tg83luc-CMVmCherry), have been described previously.^68^

#### Bacterial protein expression plasmids

pGEX-4T3-MAAP2^His^, was constructed by cloning MAAP2 ORF fused with His-tag at the C terminus in pGEX-4T3 (Cytiva).

### Lentivirus production and generation of cell lines

#### Lentivirus production

Lentiviruses were produced by transfection of 293FT cells with the expression plasmid (pTripZ or plentiCRISPRv2) and packaging plasmids (psPAX2 and pMD2.G) using PEI Max (#24765, Polysciences, Warrington, PA).^69^ At 3 dpt, the media containing the produced lentiviruses were collected and processed as described previously.^69^

#### Generation of cell lines

To generate inducible cell lines, HEK293 cells were seeded and transduced with the lentiviruses, TripZ-MAAP2-APEX2 and pTripZ-APEX2, respectively, for 3 days followed by puromycin selection. Selected cells were expanded and incubated for 1 week in puromycin-containing media. Expression of the targeted gene in inducible cells was confirmed by western blotting after doxycycline induction. The cell lines were named 293^iMAAP2−APEX2^ and 293^iAPEX2^, respectively.

To knock out the *STX7* or *SNAP23* gene, three different lentiviruses expressing distinct gRNAs were transduced simultaneously into HEK293 cells. Selected cells were expanded in puromycin-containing media for 1 week. Next, STX7-KO cells were further diluted for single-cell selection to achieve an improved knockout, whereas *SNAP23* knockout was adequate to deplete SNAP23 expression without single-cell selection. The cell lines were named STX7-KO and SNAP23-KO, respectively. Scramble single guide RNA (sgRNA)-expressing lentivirus transduced HEK293 cells were used as a control (Scramble).

### Cell viability

Gene knockout cells along with HEK293 and scramble control cells were seeded at the same density and cultured. Cells were trypsinized, stained with trypan blue (#T10282; Thermo Fisher) and counted on an automated cell counter Countess 3 (Thermo Fisher) for cell number and viability at 24, 48, and 72 h, respectively.

### Infection and transfection

#### Infection for confocal imaging

HEK293 cells were seeded on culture plates/dishes or chamber slides 1 day before infection. On the day of infection, cells were infected with wtAAV2 at a multiplicity of infection (MOI) of 5,000 viral/vector genome copies (vgc)/cell in one-fourth volume of proper culturing medium at room temperature on a rocking platform. After an hour of incubation, virus-containing media were removed, and the cells were washed twice with D-PBS (#SH30028.10, Cytiva). The cells were then replaced with fresh media followed by transfection of pHelper using PEI Max at a ratio of 1:3 (DNA:PEI Max). At 2 dpi, the cells were fixed using 4% paraformaldehyde (PFA) for immunostaining.

#### Transfection for confocal imaging

HEK293 cells were seeded on culture plates/dishes or chamber slides 1 day before transfection. On the day of transfection, pR2C2, pHelper, and prAAV2 were transfected in HEK293 cells at 1:1:1 M ratio using PEI Max at 1:3 (DNA:PEI Max) ratio. At 2 dpt, cells were fixed using 4% PFA for immunostaining.

#### Infection for APEX-mediated biotinylation

293^iMAAP2−APEX2^ and 293^iAPEX2^ cells in T25 flasks were infected with AAV2^ΔMAAP^ at an MOI of 5,000 vgc/cell. AAV2^ΔMAAP^ was unable to express MAAP2 due to the early stop codon introduced at the 19th amino acid of the protein.^11^ Protein expressions were induced at 16 h post-infection (hpi) by the addition of doxycycline (2 μg/mL). Cells were then treated for APEX2-mediated proximity labeling at 2 dpi.

### APEX2-mediated proximity labeling

APEX2-mediated proximity labeling was performed in accordance with a published protocol.^32^ Briefly, 293^iMAAP2−APEX2^ and 293^iAPEX2^ cells were infected with AAV2^ΔMAAP^ and transfected with pHelper. Doxycycline (Dox; at 2 μg/mL) was added at 16 hpi. At 48 hpi, the media of the cells were replaced with the complete DMEM media containing biotin-phenol (BP) (Biotinyl tyramide; #SML2135, MilliporeSigma) at a final concentration of 500 μM for 30 min at 37°C. Next, biotinylation was performed by adding hydrogen peroxide (H_2_O_2_, #H1009, MilliporeSigma) at a final concentration of 1 mM for 1 min. The biotinylation reaction was then quenched by aspirating media and washing cells using Quenching buffer (D-PBS) containing quenchers: 10 mM sodium ascorbate (#S1349, Spectrum Chemical, New Brunswick, NJ), 5 mM Trolox (#238813, MilliporeSigma), and 10 mM sodium azide (#014314.22, Thermo Fisher). After washing four times, cells were harvested with Quenching buffer and centrifuged at 3,000 × *g* for 10 min at 4°C. Pellets were lysed in radioimmunoprecipitation assay (RIPA) buffer (50 mM Tris-HCl, 150 mM NaCl, 0.1% SDS, 0.5% deoxycholate, 1% Triton X-100, pH 7.5) supplemented with quenchers, Protease Inhibitor Cocktail (PIC; #S8830, MilliporeSigma), and additional 1 mM phenylmethylsulfonyl fluoride (PMSF; # 786–055, G-Biosciences, St. Louis, MO). Cells were incubated on ice for 2 min, and the lysate was clarified by centrifuging at 15,000 × *g* for 10 min at 4°C. The lysates were then incubated with streptavidin magnetic beads (#88817, Thermo Fisher) on a rotator for 1 h at room temperature. Next, 90% of the lysate-bead mixtures were collected and washed twice with RIPA, pH 7.5 (without quenchers, PIC, or PMSF), once with KCl (1 M), once with Na_2_CO_3_ (0.1 M), once with urea (2 M pH 8.0), twice with RIPA (pH 8.0), and five times with TNS buffer (50 mM Tris-HCl, pH 8.0, 150 mM NaCl, 0.1% SDS). The washed beads (samples) were kept in TNS buffer and sent for qMS at the Taplin Biological Mass Spectrometry Facility, Harvard University. For the western blot analysis of the samples, 10% of the lysate-bead mixtures were washed as described above, and eluted by boiling them in 3 x Laemmli buffer (187 mM Tris-Cl, pH 6.8, 4.5%SDS, 25% glycerol, 0.015% bromphenol blue, 5% DTT) supplemented with 2 mM biotin (#B4501, MilliporeSigma), and 20 mM dithiothreitol (DTT; #DTT25, GoldBio, St Louis, MO) for 5 min at 95°C.

### On-bead digestion and liquid chromatography-tandem mass spectrometry analysis

The beads in TNS buffer were washed at least five times with 100 μL of 50 mM ammonium bicarbonate. Then, 5 μL (200 ng/μL) of modified sequencing-grade trypsin (Promega, Madison, WI) was spiked in. The samples were placed at 37°C overnight and then placed on a magnetic plate, and the supernatant was removed. The extracts were then dried in a speed-vac (∼1 h). Samples were then resuspended in 50 μL of HPLC solvent A (2.5% acetonitrile, 0.1% formic acid) and desalted by STAGE tip.^70^ On the day of analysis, the samples were reconstituted in 10 μL of HPLC solvent A. A nano-scale reverse-phase HPLC capillary column was created by packing 2.6 μm C18 spherical silica beads into a fused silica capillary (100 μm inner diameter × ∼30 cm length) with a flame-drawn tip.^71^ After equilibrating the column, each sample was loaded via a Famos auto sampler (LC Packings, San Francisco, CA) onto the column. A gradient was formed, and peptides were eluted with increasing concentrations of solvent B (97.5% acetonitrile, 0.1% formic acid). The peptides eluted were subjected to electrospray ionization and then entered a Velos Orbitrap Elite ion-trap mass spectrometer (Thermo Fisher). Peptides were detected, isolated, and fragmented to produce a tandem mass spectrum of specific fragment ions for each peptide. Peptide sequences (and hence protein identity) were determined by matching protein databases with the acquired fragmentation pattern by the software program, Sequest (Thermo Fisher).^72^ All databases include a reversed version of all the sequences, and the data were filtered to between a 1% and 2% peptide false discovery rate.

### Bioinformatic analysis of qMS data

The obtained qMS data were further analyzed based on unique peptide reads and intensity of individual peptides (Table S1). The proteins with a sum of unique reads of ≥15 (with a minimal 1 read in all three repeats) were analyzed to calculate −log_10_ *p* values (significance) using t test^73^^,^^74^ (unpaired, two-tailed) and log_2_ fold-change (FC) from the intensities of the detected peptides, which were plotted using Prism 10 (GraphPad). For functional classification, the highly enriched proteins of -log_10_ *p* > 2 and log_2_ FC > 4 were further sorted based on Gene Ontology (GO) using PANTHER v19.0.^75^^,^^76^

### Co-immunoprecipitation assay

HEK293 cells were transfected with pCI-MAAP2^Flag^ and the vector control, pCI-empty. At 2 dpt, the cells were harvested and lysed in ice-cold lysis buffer (50 mM Tris-HCl, pH 8.0, 150 mM NaCl, 1% NP-40, and PIC) on a rotator for 30 min at room temperature. Lysates were clarified by centrifugation at 12,000 × *g* for 15 min at 4°C. The supernatant was collected and incubated with 20 μL of prewashed anti-Flag-conjugated magnetic beads (#HY-K0207, MedChemExpress, Monmouth Junction, NJ) with rotation overnight at 4°C. The beads were then collected against the side of the tube by placing them on a magnetic stand and washed with Washing buffer (50 mM Tris-HCl, pH 7.4, 150 mM NaCl, 0.5% Tween 20) four times. The captured proteins were eluted using acidic elution buffer (0.15 M Glycine, pH 2.5–3.1) after 10-min incubation at room temperature. Supernatants were transferred to new tubes by collecting the magnetic beads to the side of the tubes using a magnetic stand. The acidic condition of the supernatants was neutralized using neutralization buffer (1M Tris-HCl, pH 8.0) followed by western blotting.

### *In vitro* pulldown assay

An expression plasmid, pGEX-4T3-MAAP2^His^, was transformed into BL21/DE3 pLysS *E. coli* bacteria (#L1195, Promega). Recombinant GST-fused MAAP2^His^ (GST-MAAP2^His^) was expressed and purified as previously described.^63^^,^^77^ Purified human STX7 (#14692-H07H) and SNAP23 (#pro-659) proteins were purchased from Sino Biological (Wayne, PA) and Prospec Bio (East Brunswick, NJ), respectively.

Bait protein, GST-MAAP2^His^ (∼2 μg), was mixed with the prey protein, STX7 or SNAP23 (∼2 μg), and rotated for 4 h in Binding buffer (25 mM Tris, pH 7.4, 150 mM NaCl, 1 mM EDTA, and 1% NP-40) at 4°C. Meanwhile, glutathione agarose resins (#16100, Thermo Fisher) were prewashed followed by blocking with 3% BSA-PBS for 3 h. The bait-prey mixture was then incubated with prewashed-blocked glutathione agarose resins for 3 h at 4°C with rotation. The beads were then washed three times with Washing buffer (25 mM Tris-HCl, pH 7.4, 150 mM NaCl, 0.5% NP-40). The captured proteins were then eluted by boiling in 2 × Laemmli buffer (125 mM Tris-Cl, pH 6.8, 3% SDS, 16.5% glycerol, 0.01% bromphenol blue, 3.3% DTT) at 95°C for 5 min and analyzed using Western blotting.

### rAAV production

rAAV vectors were produced following our previously published protocol.^78^^,^^79^ In brief, parent WT, Scramble (scramble sgRNA control), STX7-KO, and SNAP23-KO HEK293 cells were seeded in 100-mm dishes for small-scale or 150-mm dishes for large-scale rAAV production. Cells were triple transfected with pRepCap (R2C1, R2C2, R2C5, or R2C9), pHelper, and prAAV2 at a molar ratio of 1:1:1 using PEI Max at a ratio of 1:3 (DNA:PEI Max). One 100-mm dish of each cell line was used to produce individual rAAV (rAAV1, rAAV2, rAAV5, rAAV9) in small-scale productions. Four 150-mm dishes of each cell line were used to produce rAAV vectors in large-scale production. Briefly, cell media and pellet were harvested at 3 dpt. Media were transferred in a new tube following centrifugation (3,000 rpm for 15 min). Cell pellets were resuspended in 10 mM Tris (pH 8.0). Media were precipitated in 8.5% PEG-6000 (#A17541, Thermo Fisher Scientific Inc, Waltham, MA) and ∼3% NaCl overnight at 4°C. Precipitates then were resuspended in a buffer (150 mM NaCl, 20 mM Tris, pH 8.0). Media precipitates and cell pellets were exposed to a freeze-thaw cycle, and then sonicated. Next, the lysates were treated with deoxycholate (10%) and DNase I (4 mg/mL). The lysates were clarified by addition of CsCl, followed by CsCl ultracentrifugation. Finally purified vectors were dialyzed against PBS buffer. The yield of the produced rAAV vectors in the crude lysate (in small-scale) and/or after CsCl purification (in large-scale) was quantified by qPCR using an mCherry-specific probe (5′- FAM TTC AAG TGG GAG CGC GTG ATG AA-3′IABKFQ) as previously described.^68^

### Transmission electron microscopy

Purified vectors were negatively stained as follows. Virus samples were adhered to glow discharged carbon-film-coated 300-mesh copper grids. Grids were washed on a series of 6 droplets of water and stained with 1% uranyl acetate for 4 s, dried, and viewed in a JEOL JEM-1400 TEM at 100KV.

### rAAV transduction and transgene expression assays

HEK293 cells were transduced with rAAV5 vectors purified from the media or pellet of Scramble, STX7-KO, or SNAP23-KO HEK293 cells at an MOI 10,000 vgc/cell. At 3 dpt, cells were harvested and treated to measure luciferase activity.

Firefly luciferase activity was detected using the Luciferase Assay System (#E4550; Promega) according to the manufacturer’s instructions. mCherry expression in cells was imaged under the ZOE Fluorescent Cell Imager (Bio-Rad) at 2 dpt as indicated in the figure legends. The intensity of mCherry expression was quantified by ImageJ.^80^

### SDS-PAGE and western blotting

For SDS-PAGE, lysed cells or treated proteins, as indicated, were separated in Tris-Glycine precast gel (#NN10-816, #NB10-816, and #NB10-420, NuSep Inc, Germantown, MD) with a protein ladder (#P008, GoldBio). After transferring the proteins on a polyvinylidene difluoride (PVDF) membrane (#IPVH00010, MilliporeSigma), the membrane (blot) was blocked in 5% non-fat dry milk containing (TBS-T, 20 mM Tris-HCl, pH7.6, 150 mM NaCl, and 0.1% Tween 20) for 1 h. The blot was then incubated with an antibody diluted in 1% non-fat dry milk containing TBS-T overnight. After washing, the blot was incubated with a near-infrared (NIR) fluorescent dye-conjugated or HRP-conjugated secondary antibody for 1 h and visualized on an Odyssey imaging system (LI-COR Biotechnology, Lincoln, NE) or on a Cytiva ImageQuant 800, respectively.

### Immunostaining and confocal imaging

After 15-min fixation, infected (wtAAV2) or co-transfected cells (R2C2, pHelper, and prAAV2) were permeabilized (0.5% Triton X-100) for 5 min. Then, the cells were incubated with the first antibody for 1 h and the second antibody for another hour, after blocking for 30 min with 2% BSA. Between each treatment step, cells were washed with PBS for 5 min. Prepared slides were observed and imaged under a Leica TCS SP8 STED 3× Super Resolution Microscope. Images were processed with LAS X Life Science Microscope Software (Leica).

### Antibodies used in the study

#### First antibodies

An anti-MAAP2 antibody was produced by immunization of purified GST-fused MAAP2 in rats and verified previously.^11^ An anti-Flag (#200-301-B13) was purchased from Rockland (Limerick, PA). An anti-GST (#AE001) and an anti-β-actin (#AC026) were purchased from AbClonal (Woburn, MA). An anti-V5 (#R960-25) was purchased from Invitrogen (Carlsbad, CA), and anti-STX7 antibodies were purchased from AbClonal (#A8057) and Bethyl Laboratories (#A304-512A; Montgomery, TX) for immunostaining and immunoblotting, respectively. An anti-SNAP23 (#10825-1-AP) was purchased from Proteintech (Rosemont, IL).

#### Secondary antibodies

For Western blotting, anti-Rat DyLight 800 (#SA5-10024) was purchased from Invitrogen. Anti-rabbit DyLight 800 (#5151S) and anti-mouse DyLight 800 (#5257S) were purchased from Cell Signaling (Danvers, MA). An anti-Rabbit, HRP antibody (#111-036-047) and an Alexa Fluor 680-conjugated streptavidin antibody (#016-620-084) were purchased from Jackson Immunoresearch Laboratories Inc (West Grove, PA). An anti-mouse, HRP antibody (#A16084) was purchased from Thermo Fisher Scientific Inc. For confocal imaging, both Alexa Fluor 488-conjugated anti-rat (#A48262) and Alexa Fluor 594-conjugated anti-rabbit (#A32754) were purchased from Invitrogen. An Alexa Fluor 647-conjugated anti-rabbit (#711-606-152) was purchased from Jackson Immunoresearch Laboratories Inc.

### Statistical analysis

Prism 10 (GraphPad) was used for statistical analysis. Means and standard deviations (SDs) were generated from data of at least three independent experiments (*n* ≥ 3). Statistical significances (*p* value) were determined by Student’s t test (n.s. denotes no statistically significant difference; ∗*p* < 0.05; ∗∗*p* < 0.01; ∗∗∗*p* < 0.001; and ∗∗∗∗*p* < 0.0001).

## Data and code availability

All data needed to evaluate the conclusions in the paper are present in the paper and/or the Supplementary Materials.

## Acknowledgments

The study was supported by 10.13039/100000002NIH grants AI150877, AI156448, AI171265, and HL174593. We are grateful to the Confocal Microscopy Core Laboratory of The University of Kansas Medical Center. The Leica SP8 STED confocal microscope was supported by NIH S10 OD 023625. Cagla AKSU KUZ was supported by the Republic of Türkiye Ministry of National Education Graduate Studies Fellowship. The funders had no role in study design, data collection and interpretation, or the decision to submit the work for publication.

## Author contributions

Conceptualization, C.A.K. and J.Q.; investigation, C.A.K.; methodology, K.N., S.H., S.M., X.Z., and F.C.; validation, C.A.K.; formal analysis, C.A.K., S.H., and X.Z.; writing – original draft, C.A.K.; writing – review & editing, C.A.K., K.N., S.H., S.M., F.C., and J.Q.; project administration, J.Q.; supervision, J.Q.; funding acquisition, J.Q.

## Declaration of interests

The authors declare no competing interests.

## Supplemental information

Document S1. Figures S1–S3

Table S1. List of proteins identified by label-free quantitative mass spectrometry (qMS)

Document S2. Article plus supplemental information
