# Supplementary material for: Identification of the role of SNARE proteins in rAAV vector production through interaction with the viral MAAP
Source: Mol Ther Methods Clin Dev. 2024 Dec 5;33(1):101392. doi: 10.1016/j.omtm.2024.101392 (PMC11728075; doi:10.1016/j.omtm.2024.101392)
Supplement: Document S1. Figures S1–S3 [file mmc1.pdf]

OMTM, Volume *33*

Supplemental information

Identification of the role of SNARE proteins in rAAV vector production through interaction with the viral MAAP

Cagla Aksu Kuz, Kang Ning, Siyuan Hao, Shane McFarlin, Xiujuan Zhang, Fang Cheng, and Jianming Qiu

Supplemental Materials

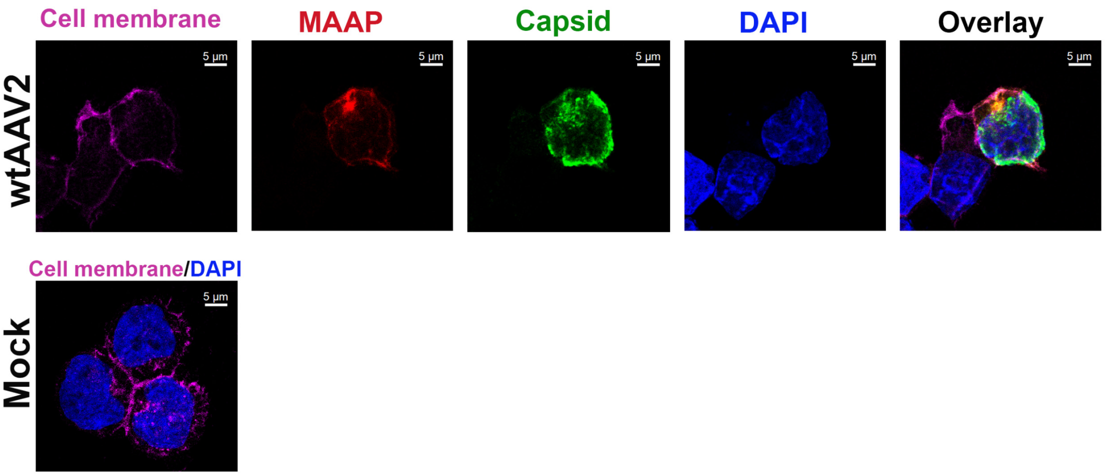

Figure S1. MAAP2 is expressed on the plasma membrane.

HEK293 cells were infected with wtAAV2 (at an MOI of 10K/cell) or mock-infected followed by pHelper transfection. At 2 dpi, live infected cells were first stained with fluorescent-conjugated cell membrane marker (#30097-T, MemBrite, Biotium). Cell membrane-stained cells were cytospun and fixed onto slides and stained for localization of MAAP2 and AAV2 capsid.

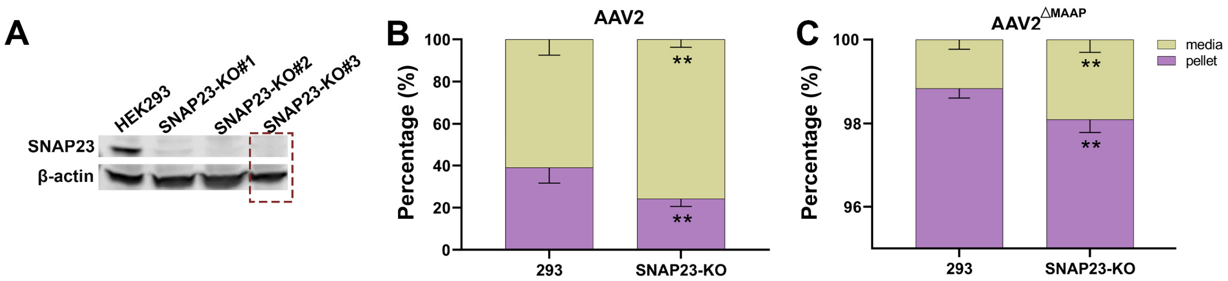

Figure S2. wtAAV2 or AAV2$^{\Delta MAAP}$ infection in SNAP23-KO cells.

(A) Generation of KO cell line. HEK293 cells were transduced with lentivirus expressing SNAP23-targeted guide RNAs (gRNA). Red, dashed-rectangle shows SNAP23-KO cells utilized in this experiment. **(B&C) Percentages of the progeny virions in the media and cells (pellet).** WT HEK293 (293) and SNAP23-KO cells were infected with wtAAV2 (B) or AAV2$^{\Delta MAAP}$ (C) followed by transfection of pHelper. At 2 dpi, the cells (pellet) and media were harvested. DNase-digestion resistant viral DNA were extracted from crude lysates of harvested pellet and media, respectively, and were quantified by qPCR using a *rep* gene probe. Bars indicate the ratios of progeny virion yields in the cells (pellet) vs in media among the total (100%). Means and standard deviations were calculated using data from three independent experiments (n=3). ** $P < 0.01$.

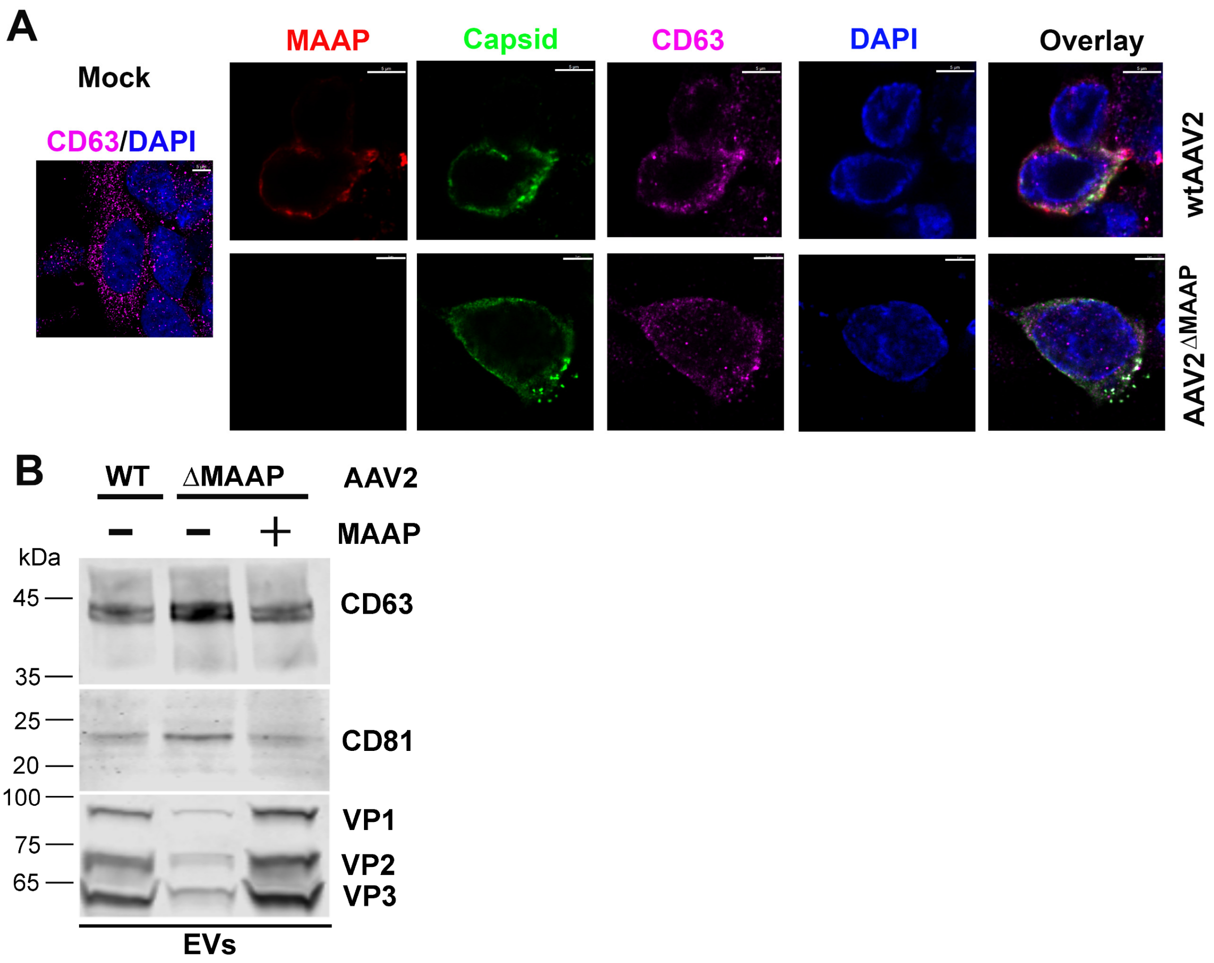

Figure S3. AAV2 capsids are associated with EVs, which is independent of MAAP expression.

(A) Immunofluorescence assay. HEK293 cells were infected with wtAAV2 or AAV2$^{\Delta MAAP}$ followed by pHelper transfection, or mock infected. At 2 dpi, infected cells were harvested and cytospun onto slides for localizations of MAAP and capsid with EV marker CD63 for exosome. Images were taken under a 100 × objective lens of a Leica STED microscope. The colors of confocal images correspond to blue for DAPI, red for MAAP, green for AAV2 intact capsids and magenta for CD63. Size bar = 5 µm. Representative confocal images are shown. **(B) Western blotting.** HEK293 cells were cultured in DMEM supplemented with 10% exosome-depleted fetal bovine serum (#A2720803, Invitrogen), and infected with wtAAV2 or MAAP-KO mutant (AAV2$^{\Delta MAAP}$), followed by co-transfection of pHelper with pCI-empty (–; for WT and AAV2$^{\Delta MAAP}$) or pCI-MAAP (+; for AAV2$^{\Delta MAAP}$), as indicated. At 2 dpi, media of the infected cells were harvested for isolation of EVs using with Total Exosome Isolation Reagent (#4478359, Invitrogen) according to the manufacturer's instructions. The EVs were immunoblotted for exosome markers, CD63 and CD81, and AAV2 capsid proteins, respectively.

Table S1. List of proteins identified through label-free quantitative mass spectrometry (qMS).

The table includes the protein names (references), gene symbols, annotations, molecular weights (MW), the number of identified peptides (reads), and their sum intensities. Additionally, it presents the calculated P values and fold-changes between the samples pulled down from MAAP2-APEX2-expressing cells and the controls from APEX2-expressing cells.
